# Clinical utility of FDG PET in Parkinson’s disease and atypical parkinsonism associated with dementia

**DOI:** 10.1007/s00259-018-4031-2

**Published:** 2018-05-19

**Authors:** Zuzana Walker, Federica Gandolfo, Stefania Orini, Valentina Garibotto, Federica Agosta, Javier Arbizu, Femke Bouwman, Alexander Drzezga, Peter Nestor, Marina Boccardi, Daniele Altomare, Cristina Festari, Flavio Nobili, for the EANM-EAN Task Force for the recommendation of FDG PET for Dementing Neurodegenerative Disorders

**Affiliations:** 1 Division of Psychiatry, University College London, London, UK; 2 St Margaret’s Hospital, Essex Partnership University NHS Foundation Trust, Epping, CM16 6TN UK; 3 Alzheimer Operative Unit, IRCCS S. Giovanni di Dio, Fatebenefratelli, Brescia, Italy; 4 Division of Nuclear Medicine and Molecular Imaging, Department of Medical Imaging, University Hospitals of Geneva, Geneva University, Geneva, Switzerland; 5 Neuroimaging Research Unit, Institute of Experimental Neurology, Division of Neuroscience, San Raffaele Scientific Institute, Vita-Salute San Raffaele University, Milan, Italy; 6 Department of Nuclear Medicine, Clinica Universidad de Navarra, University of Navarra, Pamplona, Spain; 7 Department of Neurology & Alzheimer Center, Amsterdam Neuroscience, VU University Medical Center, Amsterdam, The Netherlands; 8 Department of Nuclear Medicine, University Hospital of Cologne, University of Cologne and German Center for Neurodegenerative Diseases (DZNE), Cologne, Germany; 9 German Center for Neurodegenerative Diseases (DZNE), Magdeburg, Germany; 10 Queensland Brain Institute, University of Queensland and the Mater Hospital, Brisbane, Australia; 11 LANVIE (Laboratoire de Neuroimagerie du Vieillissement), Department of Psychiatry, University of Geneva, Geneva, Switzerland; 12 LANE – Laboratory of Alzheimer’s Neuroimaging & Epidemiology, IRCCS S. Giovanni di Dio, Fatebenefratelli, Brescia, Italy; 13 Department of Molecular and Translational Medicine, University of Brescia, Brescia, Italy; 14 Department of Neuroscience (DINOGMI), University of Genoa & Clinical Neurology Polyclinic IRCCS San Martino-IST, Genoa, Italy

**Keywords:** FDG PET, Parkinson’s disease, Progressive supranuclear palsy, Prodromal PD, Corticobasal syndrome, Corticobasal degeneration

## Abstract

**Purpose:**

There are no comprehensive guidelines for the use of FDG PET in the following three clinical scenarios: (1) diagnostic work-up of patients with idiopathic Parkinson’s disease (PD) at risk of future cognitive decline, (2) discriminating idiopathic PD from progressive supranuclear palsy, and (3) identifying the underlying neuropathology in corticobasal syndrome.

**Methods:**

We therefore performed three literature searches and evaluated the selected studies for quality of design, risk of bias, inconsistency, imprecision, indirectness and effect size. Critical outcomes were the sensitivity, specificity, accuracy, positive/negative predictive value, area under the receiving operating characteristic curve, and positive/negative likelihood ratio of FDG PET in detecting the target condition. Using the Delphi method, a panel of seven experts voted for or against the use of FDG PET based on published evidence and expert opinion.

**Results:**

Of 91 studies selected from the three literature searches, only four included an adequate quantitative assessment of the performance of FDG PET. The majority of studies lacked robust methodology due to lack of critical outcomes, inadequate gold standard and no head-to-head comparison with an appropriate reference standard. The panel recommended the use of FDG PET for all three clinical scenarios based on nonquantitative evidence of clinical utility.

**Conclusion:**

Despite widespread use of FDG PET in clinical practice and extensive research, there is still very limited good quality evidence for the use of FDG PET. However, in the opinion of the majority of the panellists, FDG PET is a clinically useful imaging biomarker for idiopathic PD and atypical parkinsonism associated with dementia.

## Introduction

There are no clear diagnostic guidelines on the role of FDG PET in the diagnostic work-up of cognitive disorders and dementia. Therefore, the European Association of Nuclear Medicine (EANM) and the European Academy of Neurology (EAN) came together in a joint initiative to provide guidance to clinicians on the use of FDG PET in the context of neurodegenerative diseases. The initiative included a set of 21 clinical scenarios, captured as PICO (Population, Intervention, Comparison, Outcome) questions, that were addressed based on literature evidence and expert consensus [1].

In this article, we focus on the assessment of the quality of studies investigating the clinical utility of FDG PET for identifying patients with Parkinson’s disease (PD) who are at risk of cognitive decline, and the utility of FDG PET in facilitating the differential diagnosis of common forms of parkinsonism, i.e. idiopathic PD, including the prodromal stage, progressive supranuclear palsy (PSP) syndromes, and corticobasal syndrome (CBS). Multiple system atrophy (MSA), another neurodegenerative disorder presenting with parkinsonism was not included in this review. MSA very seldom, if ever, affects cognition and therefore it did not fall within our prespecified condition restricting this review to studies on the role of FDG-PET in “the diagnostic work-up of cognitive disorders and dementia”.

### Parkinson’s disease

PD is a common degenerative disorder. It is associated with an increased incidence of cognitive impairment and dementia. The pathology underlying this cognitive decline is variable: while in some patients it is purely Lewy body pathology, in many it is due to mixtures of amyloid, tau and Lewy body pathology [2]. Any therapeutic intervention to stop cognitive decline is likely to be most effective in the early stages of PD or even during prodromal stages of PD. Consequently, it may become important to identify patients at high risk of cognitive decline before its onset. Older age, scores from nonmotor assessments, reduced dopamine transporter uptake in the caudate, deficit on smell testing, CSF amyloid β (Aβ42) to t-tau ratio, and APOE ε4 status are all known risk factors for cognitive decline in patients with newly diagnosed PD [3]. Here we report on the role of FDG PET in predicting cognitive decline in PD.

Not all patients presenting with parkinsonism have idiopathic PD. There are alternative pathologies that can present with parkinsonism and cognitive decline. Both PSP and CBS can mimic PD in early stages and are particularly difficult to diagnose in prodromal stages.

### Progressive supranuclear palsy

The underlying neuropathology of PSP is a characteristic four-repeat tau neuropathology [4]. There are a number of clinical phenotypes of PSP which consist of different combinations of motor, gait, language, cognitive and behavioural features [5]. The two most common phenotypes are PSP Richardson’s syndrome (PSP-RS) and the clinical phenotype that most closely mimics idiopathic PD, PSP-parkinsonism (PSP-P) which often presents with asymmetrical tremor, bradykinesia and rigidity. An initial positive response to levodopa treatment can be misleading. Frequently only later in the disease course, when patients develop additional features of impaired ocular movements with vertical supranuclear gaze palsy, is a retrospective diagnosis of PSP-P made.

### Corticobasal syndrome

CBS is an atypical parkinsonian syndrome which consists of dystonia, rigidity, akinesia, myoclonus, tremor and poor response to levodopa. Typically, there is quite marked asymmetry, including limb apraxia and the alien limb phenomenon. Other features include speech and language impairment and cognitive decline. The term CBS describes a clinical phenotype which has been shown to have a heterogeneous underlying pathology. Corticobasal pathology is found only in about 50% of all clinically diagnosed patients. This has led to the distinction between the clinical syndrome (CBS) and the pathological diagnosis (corticobasal degeneration, CBD). The other pathologies found at autopsy include Alzheimer’s disease (AD), PSP and other tauopathies, dementia with Lewy bodies and Creutzfeldt-Jakob disease. Based on this background, three literature searches were performed to assess the quality of evidence supporting the use of FDG PET in facilitating the diagnosis of PD and atypical parkinsonism associated with dementia where the underlying pathology is PSP or CBD.

## Methods

Seven panellists, four from the EANM and three from the EAN, were appointed to produce recommendations, taking into consideration the incremental value of FDG PET as an add-on investigation to a comprehensive clinical/neuropsychological assessment, in facilitating the diagnosis and management of patients with parkinsonism. Consensus recommendations were reached through a Delphi procedure which was based on the expertise of the panellists. The panellists were provided with comprehensive data regarding the availability and quality of evidence, which was assessed by an independent methodology team, as described by Boccardi et al. [6]. Briefly, we searched the literature using harmonized PICO (Population, Intervention, Comparison, Outcome) questions. Thematic keywords were generated by experts, and studies were selected based on eligibility criteria described elsewhere [6]. Relevant data were extracted from selected studies and assessed for quality of methodology, according to European Federation of Neurological Societies guidance [7] and for their relevance to FDG PET studies [6].

### PICO questions for this review

In this review, the PICO questions asked whether “performing FDG PET would add diagnostic value (in terms of increased accuracy compared with neuropathological diagnosis, biomarker-based diagnosis or diagnosis at follow-up) to standard clinical/neuropsychological assessment alone”, to:

- Identify brain dysfunction related to cognitive deterioration in patients with PD and cognitive impairment (PICO question 12)
- Discriminate PSP from PD (PICO question 13)
- Identify the underlying pathological process in patients with CBS (PICO question 15).

### Eligibility criteria

Only original full papers published in English in international journals with an impact factor were considered. Reviews, management guidelines, abstracts and ‘grey’ literature were excluded. Any sample size was permissible if pathology was the gold standard for diagnosis. Otherwise, the minimum sample size was five for PSP and CBS, and 20 for PD.

### Literature search

Electronic searches were performed using a harmonized keywords string based on the specific PICO question, and included a selection of terms chosen for being largely inclusive to identify a broad range of papers. The strings contained a common part for FDG PET and a PICO question-specific part [6]. The MEDLINE, Embase and Google Scholar databases were searched for studies published up to November 2015. The first screening of all included studies was performed by the panellist responsible for that PICO question (an expert neurologist, psychiatrist or nuclear physician) who could include additional studies based on personal knowledge or forward tracking from the references of papers. The full text of these potentially eligible studies was then independently assessed for eligibility by the methodology team.

### Data extraction and quality assessment

To assess the evidence, data on study features, population of interest, index test, gold/reference standard, and critical/proxy outcomes were extracted (for more details see, Boccardi et al. [6]). Data assessors for this review were D.A. for PICO question 12, and F.G. and S.O. for PICO questions 13 and 15. Critical outcomes were the validated measures of test performance accuracy, sensitivity, specificity, AUC, positive and negative predictive values (PPV, NPV) and likelihood ratios (LR). Additional proxy outcomes for PICO questions 13 and 15 were accuracy of differential diagnosis between typical and atypical parkinsonism. The prediction of years of survival was an additional outcome specific to PICO question 15.

The quality of evidence was consensually assessed by the methodology team based on the study design, gold/reference standard, FDG PET image assessment method (visual or semiquantitative), risk of bias, index test imprecision, applicability, effect size, and effect inconsistency. A final assessment of the relative availability of evidence was formulated, taking into account evidence available from all 21 PICO questions. This ranking was summarized as ‘very poor/lacking’, ‘poor’, ‘fair’ or ‘good’. For further details about data extraction and quality assessment see Boccardi et al. [6].

## Results

**Fig. 1:**
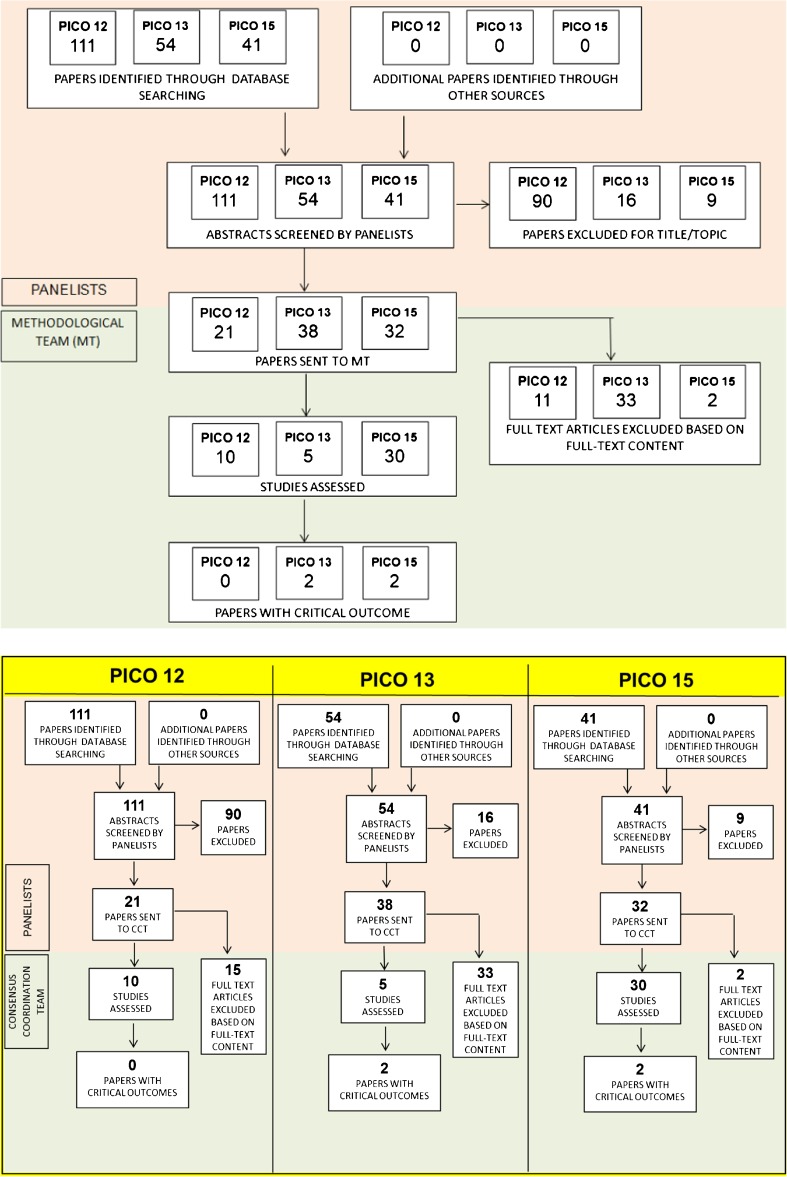
PRISMA flow chart of selected studies to identify brain dysfunction related to cognitive deterioration (PICO question 12), to discriminate PSP from PD (PICO question 13), and to identify the underlying pathological process in patients with CBS (PICO question 15) [8]

Among the three PICO questions included in this review, only four of the 91 studies examined included the critical outcomes needed for the three questions of interest (Fig. 1). Our data extraction and assessment failed to find definitive evidence for the clinical utility of FDG PET for identifying neurodegeneration associated with cognitive decline in PD and for discriminating PSP from PD. However, we ranked the evidence supporting the clinical use of FDG PET for discriminating between different underlying pathologies in patients with CBS as “fair” (see Table 1, and Nobili et al. [1]).

**Table 1:** Availability of evidence and panellists’ decisions supporting the use of FDG PET in the diagnostic work-up of the main forms of parkinsonism

PICO question	Relative availability of evidence	Panellists’ recommendations	Main reasons for final decision
12 (PD-related decline)	Very low/lacking	Yes	Sensitive to cortical involvement before cognitive deficits appear
13 (PSP)	Very low/lacking	Yes	Presence of typical hypometabolic patterns
15 (CBS)	Fair	Yes	Presence of typical hypometabolism

Delphi decisions for the other PICO questions of the EANM/EAN initiative led to supporting FDG PET in all clinical scenarios [1] with the exception of preclinical cases [9] and of amyotrophic lateral sclerosis and Huntington’s disease [10]

### PICO question 12: Identify PD-related neurodegeneration associated with impaired cognition

Of the 111 studies identified and screened by the leading panellist (Z.W.), 21 were sent to the methodology team for data extraction and assessment (see Fig. 1). Eleven studies were excluded for the following reasons: seven [11–17] provided no critical outcomes or did not have the minimum sample size, two [18, 19] did not include the target population (i.e. PD patients without cognitive impairment), in one [20] the index investigation was proton magnetic resonance spectroscopy, and one [21] focused on cognitive impairment and cerebral hypometabolism in PD patients with mild cognitive impairment (PD-MCI) with and without visual hallucinations. The table showing the full data extraction is available at: https://drive.google.com/file/d/0B0_JB3wzTvbpUHRSZ2NfSVZfVkE/view?usp=sharing.

The critical outcomes were not available in any of the examined studies. The metabolic patterns associated with PD-related cognitive impairment (i.e. MCI or dementia) as compared with the patterns in patients with no PD-related cognitive impairment were described by Yong et al. [22]. They showed that in comparison with PD patients with no cognitive impairment, patients with PD dementia (PDD) showed greater metabolic deficits in the parietal and frontal regions but that metabolic patterns in PDD patients and patients with Lewy body dementia were broadly similar.

Two longitudinal studies did identify specific patterns of hypometabolism in PD patients who developed cognitive decline during follow-up compared with the patterns in PD patients who did not develop cognitive impairment. In the first study, 6 of 23 patients with PD developed dementia during follow-up. Patients with progression to PDD had decreased metabolism in the visual association cortex, posterior cingulate cortex and caudate nucleus at baseline (at a time when no major cognitive impairment was present). With progressive impairment, the hypometabolism became more widespred widely to the neocortical regions [23]. In the second study, PD patients who declined had hypometabolism in the posterior and temporal areas of the brain at baseline, in contrast to PD patients with stable cognition over time [24]. However, a quantitative assessment of discrimination accuracy was not provided. Three further studies [25–27] analysed data from the same sample of patients and showed decreased prefrontal and parietal metabolism in PD-MCI patients relative to PD patients with no cognitive impairment, as well as an increase in brainstem/cerebellar metabolism.

**Fig. 2:**
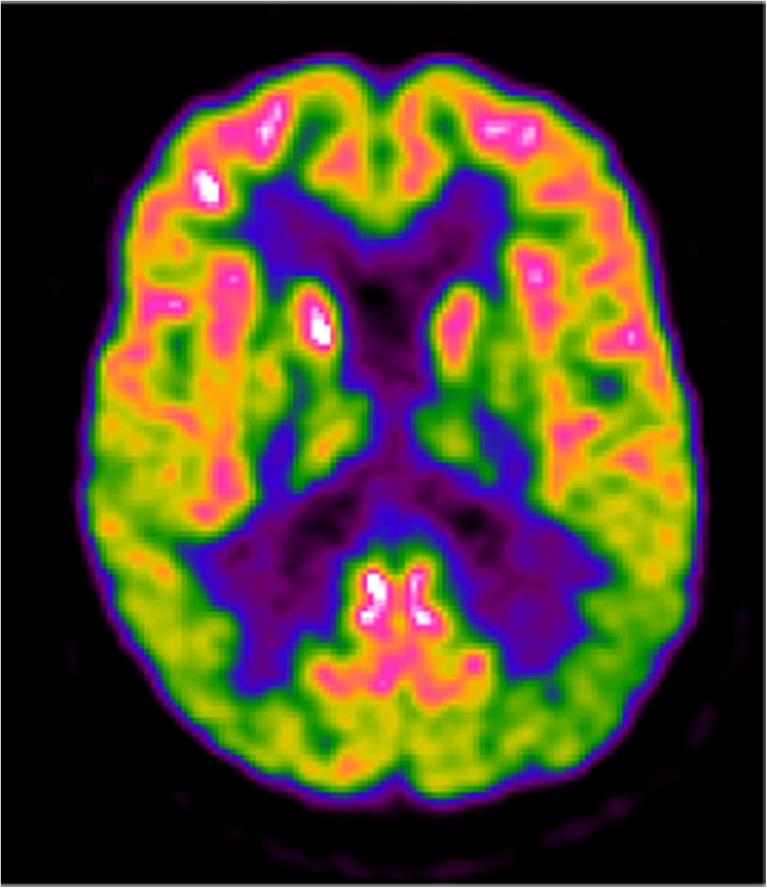
A 67-year-old patient with de novo PD and subtle cognitive impairment (amnestic MCI), MMSE score 29/30 and high educational level. The FDG PET image shows bilateral posterior parieto-occipital hypometabolism with sparing of the posterior cingulate cortex

The availability of formal evidence supporting the utility of FDG PET for identifying PD-related neurodegeneration in patients with PD at risk of cognitive impairment was therefore ‘lacking’. Nevertheless, the consensus-based recommendation was achieved on Delphi round IV, with five panellists voting for the clinical use of FDG PET based on the presence of cortical hypometabolism in PD patients prior to the onset of cognitive decline (Fig. 2).

### PICO question 13: Discriminate PSP from PD

Of 54 studies identified and screened by the assigned panellist (F.N.), 38 were sent to the methodology team (see Fig. 1), and of these 33 were excluded: 20 studies did not include PSP [28] or PD patients [29–47], 5 studies did not directly and exclusively compare PSP and idiopathic PD [38, 48–51] and the remaining 7 studies [52–58] reported associations only. The data extraction table is available at: https://drive.google.com/file/d/0B0_JB3wzTvbpNnEwTmRzX3pmdlU/view?usp=sharing). Critical outcomes were available in two of the examined studies (see Table 2), which included 36 patients with PSP and 32 with idiopathic PD. In these studies FDG PET was able to discriminate PSP from PD with sensitivities of 52.9–75%, specificities of 80–100% and accuracies of 67.6–83.9% [59, 60], and an AUC of 0.80 [60].

**Table 2:** PICO question 13: Discriminate PSP from idiopathic PD. Quality of evidence for each critical outcome

Critical outcomes	Number of studies	Sample size	Gold/reference standard	FDG PET assessment	Risk of bias^a^	Index test methods^b^	Applicability^c^	Effect (CI)^d^	Effect assessment^e^	Effect inconsistency^f^	Outcome quality^g^
Sensitivity	2	36 PSP 32 IPD	1 study Follow-up diagnosis + 1 study Clinical diagnosis	Semiquantitative	Serious	Serious	Serious	52.9% (28–77%) to 75% (49–91%)	Low	Serious	Very low
Specificity	2	36 PSP 32 IPD	1 study Follow-up diagnosis + 1 study Clinical diagnosis	Semiquantitative	Serious	Serious	Serious	80% (56–94%) to 100% (73–100%)	High	Not serious	Low
Accuracy	2	36 PSP 32 IPD	1 study Follow-up diagnosis + 1 study Clinical diagnosis	Semiquantitative	Serious	Serious	Serious	67.6% (50–82%) to 83.9% (66–94%)	Moderate	Not serious	Low
AUC	1	19 PSP 12 IPD	1 study Clinical diagnosis	Semiquantitative	Serious	Serious	Serious	80% (CI NA)	Moderate	NA	Very low

Relative availability of evidence: very low/lacking

*CI* 95% confidence interval, *NA* not applicable,*PSP* progressive supranuclear palsy, *IPD* idiopathic Parkinson’s disease

^a^Assessment of the study design and other methodological features (e.g. patient selection, clinical diagnostic criteria used)

^b^Assessment of index test methodology (e.g. technical details, image analysis methods and statistical analysis)

^c^Representativeness of the studied population and index test reproducibility in clinical practice (semiquantitative methods correspond to ‘serious’ indirectness, visual + semiquantitative methods correspond to ‘not serious’ indirectness, due to partial implementation of quantitation in clinical practice)

^d^Lowest and highest values for each critical outcome; when more than one value was obtained for the same outcome, the highest was reported.

^e^Low 51–70%, moderate 71–80%, high 81–100%

^f^‘Not serious’ if difference between lowest and highest values 0–20, ‘serious’ 21–40, ‘very serious’ >40

^g^Summary of evidence from all columns

Additional outcomes were reported in three additional studies [61–63] using a two-step classification, consisting first of discriminating idiopathic PD from atypical parkinsonism, and then discriminating PSP from other atypical parkinsonism. In these studies, FDG PET demonstrated good sensitivity in discriminating idiopathic PD from atypical parkinsonism (range 83–86%) and a moderate sensitivity in diagnosing PSP and discriminating it from CBD and MSA (range 73–88%). Specificity and PPV were both greater than 90% in discriminating idiopathic PD from atypical parkinsonism (range 94%–97% and 96%–98%, respectively) and in discriminating various subtypes of atypical parkinsonism (range 90%–94% and 85%–96%, respectively). The NPV for discriminating idiopathic PD from atypical parkinsonism was moderate (range 76–83%).

The five studies had a moderate risk of bias regarding the index test. In the studies by Mudali et al. [59] and Srulijes et al. [60], the level of blindness of the FDG PET reader to the clinical diagnosis was unclear and in those by Teune et al. [57] and again Srulijes et al. [60], the execution of the index test was not described in sufficient detail to allow replication. Further, there was low applicability of all studies due to all studies having included a selected population and having used a semiquantitative method of image analysis.

**Fig. 3:**
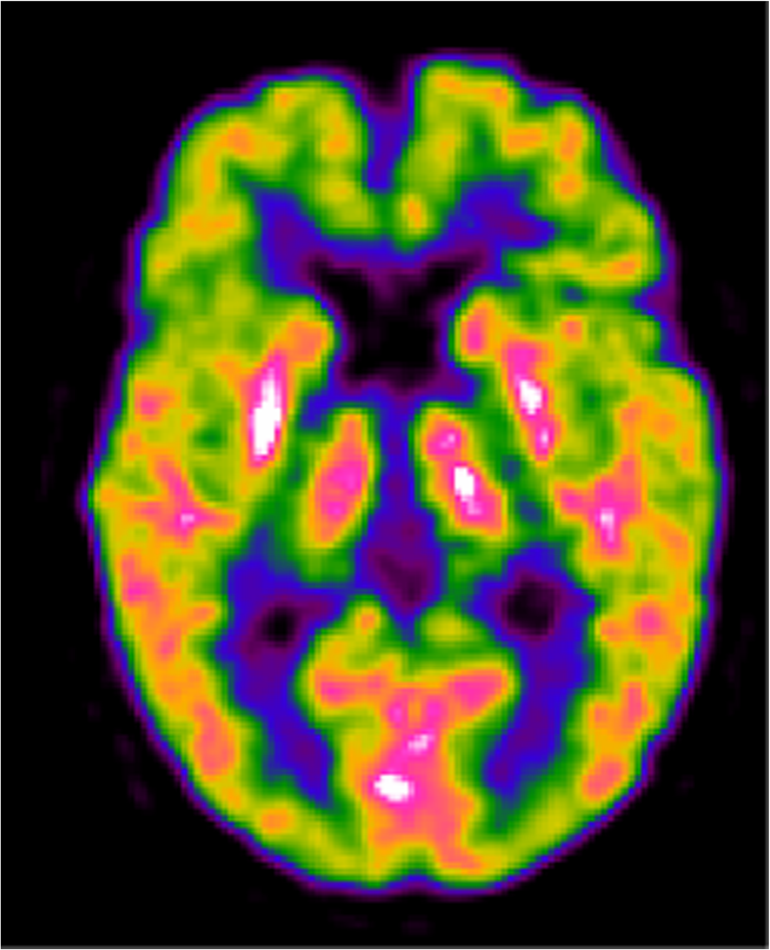
A 58-year-old patient with PSP and mild cognitive impairment (non-amnesic multiple domain MCI; MMSE score 26/30). The FDG PET image shows moderate hypometabolism of the bilateral frontal cortex, including the medial frontal gyri, and hypometabolism of the caudate nuclei mainly in the right hemisphere, and in the right thalamus

Taking into account the available evidence for the PICO questions of the entire project, the level of evidence supporting the clinical use of FDG PET for discriminating PSP from idiopathic PD was considered as ‘lacking’. However, the consensus recommendation to support the use of FDG PET was defined on Delphi round III and was based on the presence of a typical metabolic pattern of hypometabolism in PSP which predominantly affects the frontal, thalamic, striatal and midbrain regions (Fig. 3).

### PICO question 15: Identify the underlying pathological process in patients with corticobasal syndrome

Among the 41 studies identified by the assigned panellist (F.N.), 32 were sent to the methodological team (see Fig. 1). Two studies [38, 59] were excluded because they did not include CBS patients. The table reporting the data extraction is available at: https://drive.google.com/file/d/0B0_JB3wzTvbpamRDZnNpV2VQSE0/view?usp=sharing). Critical outcomes were available in two of the examined studies (Table 3). Both studies [64, 65] used amyloid-PET as the gold standard for clinical diagnosis, and tested the ability of FDG PET to predict AD pathology in a total of 39 patients with CBS. Sensitivity ranged from 91% to 95%, specificity from 58% to 75%, and accuracy from 73% to 82%. Taswell et al. [65] also reported a PPV of 68%, NPV of 97%, LR+ of 3.90 and LR− of 0.06.

**Table 3:** PICO question 15: Identify the underlying molecular pathology (e.g. amyloidosis or tauopathies) in CBS patients. Quality of evidence for each critical outcome

Critical outcome	Number of studies	Sample size	Gold/reference standard	FDG PET assessment	Risk of bias	Index test methods	Applicability	Effect (CI)	Effect assessment	Effect inconsistency	Outcome quality
Sensitivity	2	39	Biomarker-based diagnosis (amyloid-PET)	1 Visual + semiquantitative 1 Semiquantitative	Not serious	Serious	Serious	91% (59–100%) to 95% (standard error: 4%)	High	Not serious	Moderate
Specificity	2	39	Biomarker-based diagnosis (amyloid-PET)	1 Visual + semiquantitative 1 Semiquantitative	Not serious	Serious	Serious	58% (29–82%) to 75% (standard error: 14%)	Low	Not serious	Moderate
Accuracy	2	39	Biomarker-based diagnosis (amyloid-PET)	1 Visual + semiquantitative 1 Semiquantitative	Not serious	Serious	Serious	73% (51–88%) to 82% (standard error: 10%)	Moderate	Not serious	Moderate
PPV	1	14	Biomarker-based diagnosis (amyloid-PET)	1 Semiquantitative	Not serious	Serious	Serious	68% (NA)	Low	NA	Moderate
NPV	1	14	Biomarker-based diagnosis (amyloid-PET)	1 Semiquantitative	Not serious	Serious	Serious	97% (NA)	High	NA	Moderate
LR+	1	14	Biomarker-based diagnosis (amyloid-PET)	1 Semiquantitative	Not serious	Serious	Serious	3.90 (NA)	Moderate	NA	Moderate
LR-	1	14	Biomarker-based diagnosis (amyloid-PET)	1 Semiquantitative	Not serious	Serious	Serious	0.06 (NA)	High	NA	Moderate

Relative availability of evidence: fair

*CI* 95% confidence interval, *NA* not applicable

^a^Assessment of the study design and other methodological features (e.g. patient selection, clinical diagnostic criteria used)

^b^Assessment of index test methodology (e.g. technical details, image analysis methods and statistical analysis)

^c^Representativeness of the studied population and index test reproducibility in clinical practice (semiquantitative methods correspond to ‘serious’ indirectness, visual + semiquantitative methods correspond to ‘not serious’ indirectness, due to partial implementation of quantitation in clinical practice)

^d^Lowest and highest values for each critical outcome; when more than one value was obtained for the same outcome, the highest was reported.

^e^Low 51–70%, moderate 71–80%, high 81–100%

^f^‘Not serious’ if difference between lowest and highest values 0–20, ‘serious’ 21–40, ‘very serious’ >40

^g^Summary of evidence from all columns

Both studies had a low risk of bias for all items, and moderate applicability concerns. Because of the paucity of results, accuracy of differential diagnosis between CBD and idiopathic PD or other atypical parkinsonism, and prediction of years of survival were added as additional outcomes. They were extracted from 9 of the 28 remaining studies:

- Differential diagnosis between CBS and other atypical parkinsonism was described in four studies [48, 50, 51, 61]. They showed moderate-to-good sensitivity (81–91%) and very good specificity (91–100%).
- Differential diagnosis between CBD and PSP was analysed in four studies [30, 32, 41, 54]. They showed good AUC (0.92–0.97), moderate sensitivity (76–79%) and heterogeneous specificity (69–92%).
- Predictability of survival: in the study by Hellwig et al. [54], FDG PET alone was an independent predictor of survival in patients with parkinsonism (age-adjusted hazard ratio: 5.15 for PSP/CBS FDG PET diagnosis).
- Cordato et al. [66] reported similar hypometabolic patterns in autopsy-confirmed CBD and PSP.

**Fig. 4:**
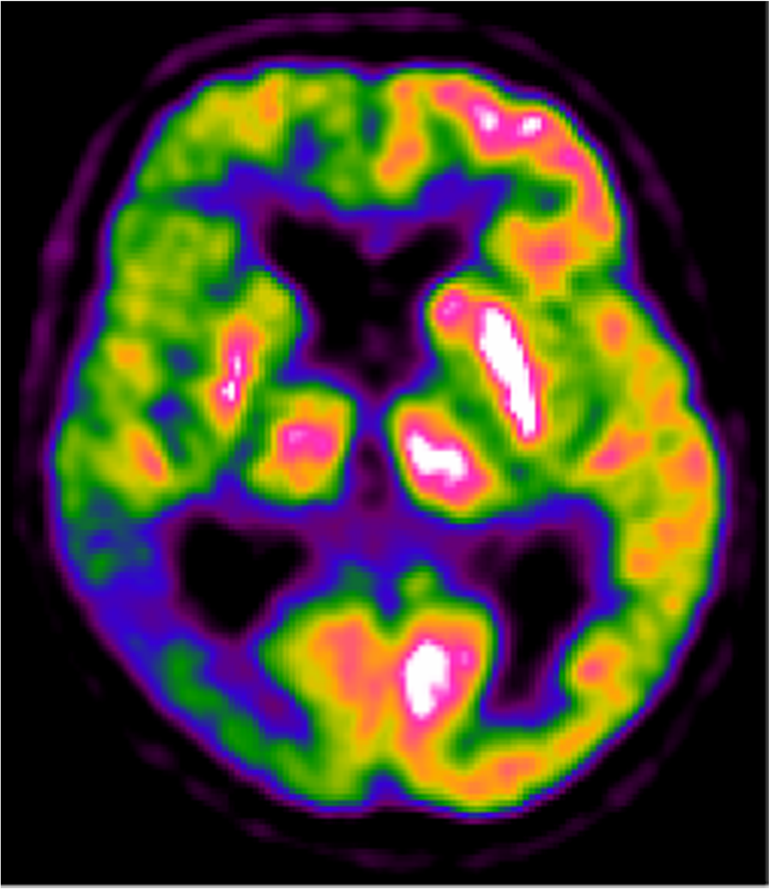
A 76-year-old patient with CBS and mild cognitive impairment (non-amnestic, multiple domain MCI; MMSE score 28/30). The FDG PET image shows severe hypometabolism in the whole right hemisphere, including the basal ganglia and thalamus, with more severe hypometabolism in the temporoparietal cortex. Dopamine transporter SPECT imaging showed severely reduced uptake in the right basal ganglia, suggesting CBD

In all of these studies, the most frequent pattern of hypometabolism associated with CBS included asymmetrical hypometabolism of the parietal and frontal cortex, thalamus and basal ganglia. The occipital cortex and cerebellum were usually spared. Considering the entire project, the availability of formal evidence supporting the clinical use of FDG PET in identifying the underlying neuropathology in CBS patients was considered as ‘fair’. Consensus was achieved on Delphi round I, with six panellists supporting the clinical use of FDG PET based on the specific asymmetry of the pattern of hypometabolism (Fig. 4).

## Discussion

We assessed the evidence for the clinical utility of FDG PET in diagnosing idiopathic PD and atypical parkinsonism associated with dementia. The evidence supporting the clinical use of FDG PET for identifying PD-related neurodegeneration associated with impaired cognition and for discriminating PSP from idiopathic PD was lacking. Conversely, the evidence supporting the clinical use of FDG PET for identifying the underlying neuropathology in patients with CBS was fair. Despite the low quality of evidence available from published studies, the Delphi panel voted for recommending the clinical use of FDG PET in the differential diagnosis of conditions characterized by parkinsonism and dementia (PICO question 12).

The reasons given by panellists during the Delphi panel process focused on both the clinical utility of the NPV of FDG PET, and also on the PPV of typical patterns of hypometabolism. In particular for the identification of impending PD-related cognitive decline, the panellists recommended the clinical use of FDG PET because patients with PDD or PD-MCI have a typical pattern of hypometabolism mainly affecting the posterior cortical areas. The prognostic value of FDG PET was also considered to be clinically relevant in the identification of patients who may benefit from early cholinesterase inhibitor treatment or other future symptomatic treatments. A recently published study [67] showed that FDG PET with statistical parametric mapping detected patterns of hypometabolism that predicted the risk of a patient with PD having progressed to dementia by 4 years with 85% sensitivity and 88% specificity. However, this study was not available at the time of the literature search and therefore was not considered in the Delphi process. Atypical hypometabolism of mainly posterior cortical areas on FDG PET could therefore be added to the list of other helpful investigations which include reduced dopamine transporter uptake in the caudate, CSF amyloid β (Aβ1-42) to t-tau ratio and APOE ε4 status [3], and is a risk factor for cognitive deterioration in idiopathic PD.

For PICO question 13, panellists based their decision to support the clinical use of FDG PET for discriminating PSP from idiopathic PD on the presence of a typical metabolic pattern for PSP, which is not present in PD. PSP is characterized by hypometabolism in the medial frontal and anterior cingulate cortices, and in the striatum and midbrain. FDG PET may therefore be useful in early stages of the disease, when the clinical diagnosis is less certain. Perfusion SPECT, albeit less precise because of poorer spatial resolution, also displays a consistent pattern [68, 69]. The described abnormalities in PSP can be difficult to detect in very early stages by visual analysis alone and a semiautomated assessment, comparing the pattern in the patient with the pattern in age-matched controls is recommended in addition to visual reading, consistent with recommendations of the present EANM-EAN initiative [70]. While a PSP-related pattern has been repeatedly demonstrated [33, 62, 71], data are still incomplete if different PSP phenotypes are considered, such as PSP-P or pure akinesia with gait freezing. These may be characterized by less severe or incomplete patterns, compared with typical and full-blown PSP. The panellists’ decision was consistent with both the EANM procedural guidelines [72] and the more recent diagnostic criteria of the Movement Disorder Society [5, 73], which support the use of FDG PET for discriminating PD from atypical parkinsonian syndromes.

Regarding CBS (PICO question 15), an asymmetrical cortical hypometabolism is known to affect the hemisphere contralateral to the side with akinetic-rigid parkinsonism and apraxia. This hypometabolism is typically found in the motor and premotor cortices, but may also involve the prefrontal or posterior parietal and lateral temporal cortex, and the cingulate gyrus. The heterogeneity of metabolic patterns found in CBS when no autopsy diagnosis is available is most likely due to the variety of different pathologies that can present as CBS. These include CBD, PSP, AD and frontotemporal dementia, and a mix of these conditions. The future challenge is to differentiate the metabolic patterns associated with different underlying pathologies and will require either neuropathological confirmation of the diagnosis by autopsy or the use of additional imaging methods, for example tau-PET and amyloid-PET as the gold standard.

Considering the large number of studies published, the fact that the majority had severe limitations and did not allow the generation of evidence that could confidently support the clinical use of FDG PET in the three PICO scenarios was striking. It is important that future studies seek to recruit sufficient numbers of patients and have a robust clinical diagnosis and follow-up and ideally include additional imaging or other biomarkers to strengthen diagnostic certainty. Studies should preferably include autopsy diagnosis as the gold standard. Other important considerations are appropriate clinical comparisons, innovative statistical analysis and the use automatic semiquantitative assessment in addition to visual assessment, to help the nuclear medicine physician interpret findings and to increase specificity and/or sensitivity. In the case of PD, analysing FDG PET using a spatial covariance method could identify characteristic patterns of metabolism in PD with and without cognitive decline. This could be helpful in future research not only to aid more accurate diagnosis but also to evaluate the effect of new therapeutic interventions [74].
